# Multifocal Multisystem Langerhans Cell Histiocytosis Involving Pituitary Masquerading as Crohn's Disease: A Case Report and Review of the Literature

**DOI:** 10.1155/2022/4672473

**Published:** 2022-12-05

**Authors:** Mohd. Ashraf Ganie, Bhanu Malhotra, Manpreet Saini, Arshiya Dutta, Atul Sharma, Kim Vaiphie, Pinaki Dutta

**Affiliations:** ^1^Department of Endocrinology, Sher-I-Kasmir Institute of Medical Sciences, Srinagar, India; ^2^Department of Endocrinology, PGIMER, Chandigarh 160012, India; ^3^Baba Farid University, Faridkot, Punjab, India; ^4^MMC and Hospital, Solan, Himachal Pradesh, India; ^5^Department of Histopathology, PGIMER, Chandigarh 160012, India

## Abstract

*Background/Objective*. We present a case of Langerhans cell histiocytosis (LCH) with gastrointestinal involvement masquerading as inflammatory bowel disease (IBD) in a patient who initially had features of central diabetes insipidus (CDI). *Case Report.* A 19-year-old male presented at 14 years of age with central diabetes insipidus. He subsequently developed panhypopituitarism and sellar-suprasellar mass, the biopsy of which was inconclusive. Secondary causes for hypophysitis were ruled out. Five years later, he developed perianal pus discharging sinuses, positive ASCA, and sacroiliitis. Rectal ulcer biopsy showed nonspecific inflammation and necrosis. Hence, he was managed as inflammatory bowel disease (IBD). Due to nonresponsiveness of symptoms, doubt about diagnosis was invoked and rectal ulcer biopsy was repeated, which then showed infiltration by Langerhans cells. Hence, he was diagnosed with LCH and showed resolution of symptoms on initiating steroids and vinblastine. *Discussion*. Gastrointestinal involvement by LCH is unusual and only rarely has represented a prominent clinical manifestation. In most cases, such involvement suggests widespread multisystem disease. Its distinctive morphologic and immunohistochemical features allow LCH to be distinguished from other inflammatory infiltrations found in mucosal biopsy specimens. *Conclusion*. Preceding CDI and hypopituitarism may predict LCH in patients with IBD-like diseases.

## 1. Introduction

Langerhans cell histiocytosis (LCH) is characterized by abnormal proliferation of Langerhans cells (antigen-presenting immune cells). The disease has characteristics of both an abnormal reactive and a neoplastic process. It is associated with mutations in BRAF V600E and MAPK pathway [1]. It may present as a rash or bony lesions or be disseminated and involve bone marrow, lungs, liver, spleen, lymph nodes, gastrointestinal (GI) tract, and the pituitary gland. Hypothalamic-pituitary region (HPR) infiltration is present in 5%–50% of the cases with LCH [2]. Central diabetes insipidus (CDI) and anterior pituitary deficiencies frequently develop in HPR-involved LCH. The most common hormone deficiency is growth hormone (GH) (53–67%) followed by gonadotropin (53–58%) and thyroid-stimulating hormone (3.9%) deficiency [3, 4]. Gastrointestinal involvement is rare in LCH (1–5%) [5]. It may be totally asymptomatic, incidentally found on colonoscopy or presented as haematochezia, diarrhoea, abdominal pain, and vomiting. Here, we present a case of LCH masquerading as IBD in a young male who initially had features of CDI.

## 2. Case Report

**Figure 1:**
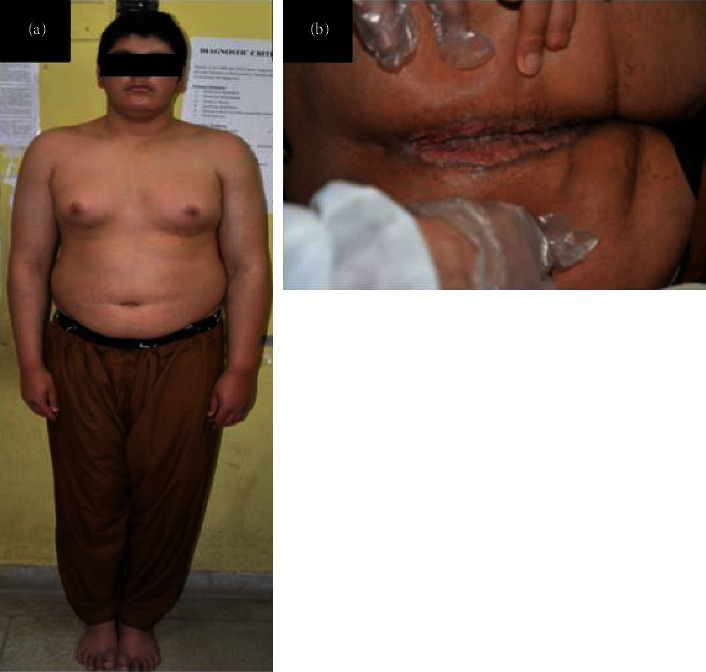
Clinical image of the patient showing (a) gynaecomastia and absent body and facial hair (b) perianal ulcers.

**Figure 2:**
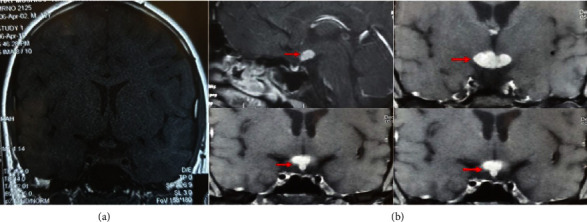
MRI sella showing (a) enhancing lesion involving hypothalamus and infundibular stalk 14 × 8 mm, isointense on T1W and hypointense on T2W at 3 years after 1^st^ presentation, and (b) increase in size of enhancing mass lesion in the upper part of stalk 20 × 17 × 15 mm, 2 years after biopsy.

A 19-year-old male, presented at 14 years of age with polyuria and polydipsia for a duration of one year. There was no history of headache, visual disturbances, or pain abdomen at that time. He was obese and had gynaecomastia (Figure 1(a)). Evaluation at the time revealed CDI on the water deprivation test. MRI sella was abnormal for loss of posterior pituitary bright spot but a normal stalk and anterior pituitary (Figure 2(a)). Anterior pituitary hormonal profile was suggestive of central hypothyroidism with normal cortisol (Total T4 = 4.87 *µ*g/dl (5–12 *µ*g/dl), 8 am Cortisol-14.6 *µ*g/dl (5–25 *µ*g/dl), LH-0.24 (1.42–15.4 IU/L), FSH-0.13 (0.3–10 IU/L), Testosterone < 10 (100–300 ng/dl). He was prescribed oral desmopressin and levothyroxine but he defaulted on medication with poor compliance.

Three years later, he had presented with delayed puberty. Repeated investigations confirmed hypogonadotropic hypogonadism and CDI (water deprivation test was repeated) along with central hypothyroidism (Total T4-5.55 *µ*g/dl (on thyroxine), 8am Cortisol-20.57 *µ*g/dl (on prednisolone), testosterone-22.1 ng/dl, LH-0.37 mIU/ml, FSH-1.93 mIU/ml). Repeated MRI showed an enhancing lesion involving the suprasellar area (hypothalamus) and thickened stalk (Figure 2(b)). Tumor markers were negative and secondary causes for hypophysitis were ruled out (Table 1). The patient was subjected to biopsy via trans-sphenoidal approach. Intraoperative findings were reported as soft, suckable, yellowish, moderately vascular, and nonencapsulated supra-sellar mass (7 × 12 × 4 mm), and a crush biopsy revealed features suggestive of an inflammatory mass. Histopathological examination (HPE) revealed features of dense chronic inflammation. Immunostaining for CD1a was inconclusive probably due to nonrepresentative sample. Postoperatively, patient was continued on oral desmopressin, prednisolone, injectable testosterone, and levothyroxine therapy.

After two and half years, patient developed painful perianal ulcers with serosanguineous discharge (Figure 1(b)). Colonoscopy revealed multiple perianal fistulae with diffuse rectal erythema. Antisaccharomyces *cerevisiae* antibody (ASCA) titre was positive (61.7 units), P-ANCA was negative, and fecal calprotectin was not performed. (Table 1). Ulcer biopsy revealed necrotic bits with slough and granulation tissue. Culture of the discharge grew *Klebsiella* pneumonia. The patient then developed pain in bilateral hip region associated with low back ache. Contrast-enhanced computed tomography (CECT) pelvis suggested features of sacroiliitis and perirectal fat stranding. In view of proctitis, sacroiliitis, and positive ASCA, the patient was diagnosed and managed with inflammatory bowel disease (IBD).

**Figure 3:**
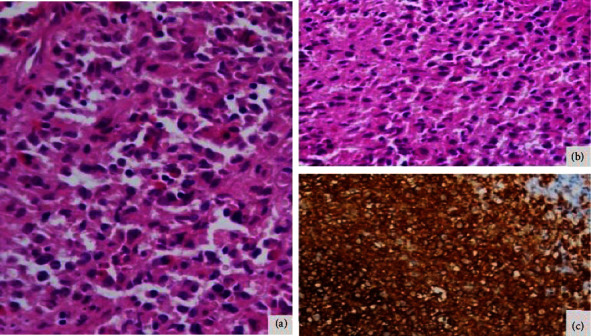
A panel of photomicrograph of perianal lesion showing sheets of epithelioid like cells with abundant amount of eosinophilic cytoplasm having mildly pleomorphic round to oval nuclei with nuclear grooving. Admixed with these cells are scattered eosinophils, lymphocytes, and plasma cells (a) (HE, x25). (b) The cell morphology is brought out better in high-power photomicrograph (HE, x400). (c) Immunohistochemical staining showing atypical histiocytic cells positive for CD1a, Langerin, and S-100 (not shown) confirming a diagnosis of Langerhans cell histiocytosis (by peroxidase anti-peroxidase, x500 on paraffin-embedded tissue sections).

Due to lack of improvement in his symptoms, a repeat biopsy was performed from the perianal lesion, which developed later in the course of illness. HPE showed sheets of epithelioid cells with abundant eosinophilic cytoplasm having pleomorphic round to oval nuclei with nuclear grooving. Immunohistochemical (IHC) studies showed atypical histiocytic cells which stained positively for CD1a, Langerin, and S-100, confirming the presence of Langerhans cells (Figure 3). After ruling out other diseases, the patient was diagnosed with LCH with pituitary and gastrointestinal involvement. He was treated with steroids and vinblastine which resulted in improvement of his symptoms. CDI was resolved and perianal ulcers were healed. Hormone replacement was still being continued.

## 3. Discussion

We present a case of a young male who had initially presented with CDI and subsequently developed panhypopituitarism, sellar-suprasellar mass lesion, and inconclusive sellar biopsy. Subsequent development of rectal ulcers with fistulae and positive ASCA suggested it to be a case of inflammatory bowel disease (IBD). ASCA seropositivity has been reported in Crohn's disease, ulcerative colitis, celiac disease, Behcet's disease, and hidradenitis suppurativa [6, 7]. It has been linked to common physiopathology such as inflammation of small intestine mucosa, loss of oral tolerance of alimentary antigen(s), altered gut permeability, and immune dysregulation (IL-23) [8]. Bilateral sacroiliitis also supported the diagnosis of IBD, though inflammatory involvement of sacroiliac joint and intervertebral disc can also occur in LCH [9]. Infundibulo-hypophysitis was assumed to be an extraintestinal manifestation of IBD. Previously, two cases of hypophysitis with CDI have been reported in IBD. Both the patients were middle-aged females (mean age-40 years) who developed sellar mass lesions several years after the diagnosis of IBD was made. While one patient had lymphocytic hypophysitis, the other had biopsy proven granulomatous hypophysitis which was refractory to steroids and responded to infliximab [10, 11].

LCH is a rare hematopoietic neoplastic disease, which can be diagnosed among all age groups; however, it is more common in children of 1–3 years of age. The incidence is three to five cases per million children, whereas in adults, it is one to two cases per million per year [12, 13]. The new disease classification has been simplified to single system LCH and multisystem LCH [14]. Multisystem LCH, seen in 45% of cases, involves two or more organs or systems. LCH has a particular predilection for involvement of the hypothalamic-pituitary axis. In a retrospective study by Kaltsas et al., out of 12 adults LCH with GI involvement, DI and anterior pituitary dysfunction were present in 3 and 4 patients, respectively, but only 1 had both at the onset. CDI can antedate, concur, or develop subsequent to the diagnosis of LCH, based on the presence of other lesions. CDI can be the initial presenting feature in about 1/3 of patients [15]. Pituitary stalk thickening can precede peripheral lesions by several months. During this early phase of the disease, a water deprivation test and possibly pituitary stalk biopsy may be helpful [16]. Among paediatric patients, multisystem disease and craniofacial involvement at diagnosis carry a significantly increased risk to develop DI during their course [17].

**Figure 4:**
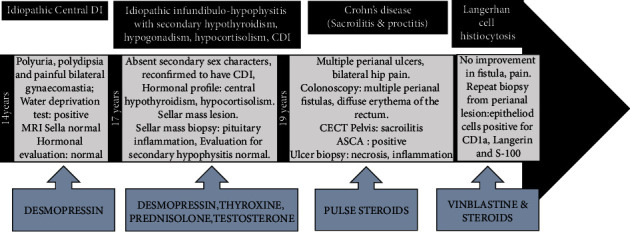
Flow chart showing the clinical course of the patient.

LCH involvement of the gastrointestinal tract (GI) is exceedingly rare. It occurs most commonly in the context of the multisystem disease. The GI manifestations could be confused with infectious, allergic, immunodeficiency, and inflammatory bowel diseases. GI-LCH is more common in children aged <2 years with a male preponderance (male : female = 2 : 1) and has poor prognosis [18]. LCH in the paediatric population classically presents with symptoms including diarrhoea, bloody stools, failure to thrive, abdominal pain, and vomiting [19]. In adults, LCH is reported as an isolated finding within the GI tract. It characteristically presents as an incidental polyp found on colonoscopy in asymptomatic individuals being evaluated for another reason [20]. Singhi et al. described 10 cases of adult LCH involving the GI tract. Five out of 10 adults were asymptomatic and the other five were presented with unrelated symptoms when the LCH was diagnosed during screening colonoscopy, and none had antecedent central diabetes insipidus. AndiónCatalán et al. [13] reported 2 paediatric patients of GI-LCH, of which one was a 12-year-old girl who was initially presented with diabetes insipidus, with subsequent development of panhypopituitarism. Further testing (colonoscopy) revealed LCH in gastrointestinal tract. Flow chart showing clinical course of the patient is shown in Figure 4.

This case highlights the varied manifestations of LCH that develop over a course of time. In this patient, it was the rectal involvement that gave insight into the disease pathology and clinched the diagnosis.

## 4. Conclusion

Preceding CDI and hypopituitarism may predict LCH in patients with IBD-like diseases.

## Figures and Tables

**Table 1:** Diagnostic evaluation for etiology of secondary hypophysitis.

Laboratory test	Result	Reference range
ESR	37 mm/hr	0–9
ANA	Negative	
Serum alpha-fetoprotein (AFP)	1.2 ng/ml	0–8.5
Serum beta hCG	<0.01 IU/l	<25
CSF alpha-fetoprotein	0.09 IU/l	
CSF beta hCG	Undetectable	<0.4 ng/ml
Serum ACE	26.0 U/L	8–65
Anti-*Saccharomyces cerevisiae* (ASCA) IgG antibody	61.70 Units	0–20
Anti-myeloperoxidase antibodies (p-ANCA)	6.51 Units	<20
